# *Notes From The Field:* Mumps Outbreak in a Recently Vaccinated Population — Kosrae, Federated States of Micronesia, August–December, 2017

**DOI:** 10.15585/mmwr.mm6804a5

**Published:** 2019-02-01

**Authors:** Susannah L. McKay, Afeke Kambui, Livinson A. Taulung, Ashley Tippins, Maribeth Eckert, Adam K. Wharton, Rebecca J. McNall, Carole Hickman, W. Thane Hancock, Carter Apaisam, Peter Judicpa, Manisha Patel, Janell Routh

**Affiliations:** ^1^Epidemic Intelligence Service, CDC; ^2^Division of Viral Diseases, National Center for Immunization and Respiratory Diseases, CDC; ^3^Pacific Island Health Officers Association, Honolulu, Hawaii; ^4^Kosrae Department of Health Services; ^5^Immunization Services Division, National Center for Immunization and Respiratory Diseases, CDC; ^6^Division of State and Local Readiness, Office of Public Health Preparedness and Response, CDC; ^7^Federated States of Micronesia Department of Health, Education and Social Affairs.

On August 6, 2017, the Kosrae Department of Health Services (KDHS) in the Federated States of Micronesia identified a confirmed case of mumps in a Kosrae resident who had 2 documented doses of measles-mumps-rubella (MMR) vaccine. The patient aged 18 years had recently traveled to Seattle, Washington, which was experiencing a mumps outbreak among members of its Pacific Islander population. Other Pacific Islands were concurrently experiencing large mumps outbreaks (*1*,*2*), in some places exceeding 500 cases, raising concern about the possibility of a similar outbreak in Kosrae. By October 6, KDHS had identified 17 cases (nine laboratory confirmed and eight suspected [clinically diagnosed as parotitis]) on the island (population 6,600) (Figure), with an attack rate of 14 cases per 1,000 residents in the primary affected municipality. At the request of KDHS, CDC deployed a team on October 17 to assist KDHS in investigation and control activities. The KDHS-CDC team conducted active surveillance to assess outbreak magnitude, interviewed mumps patients, collected specimens for laboratory testing, and reviewed patients’ vaccination records. KDHS conducted islandwide awareness campaigns about the outbreak and mumps prevention measures, and highlighted the importance of vaccination.

**FIGURE:**
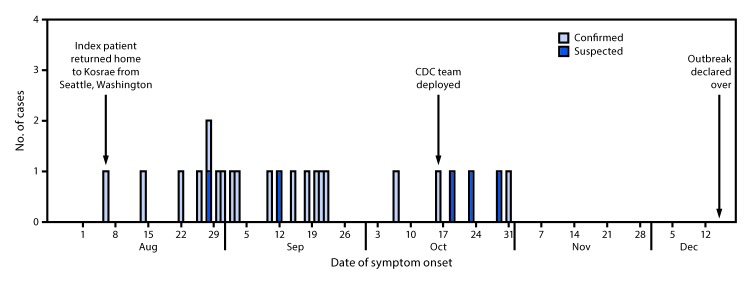
Number of suspected and confirmed mumps cases, by date of symptom onset — Kosrae, Federated States of Micronesia, August–December, 2017

By December 15, a total of 23 mumps cases with onset dates August 5–November 1, 2017, had been identified; 52% of patients were male, and the median age was 14 years (range = 1–26 years). Common symptoms reported were parotitis (20 patients; 95%), fever (20; 95%), and headache (18; 86%); one young patient was hospitalized. Seven patients (30%) reported contact with the index patient, and epidemiologic links established for 20 patients showed that transmission occurred primarily via the island’s school system. Twenty-one (91%) patients had received the recommended ≥2 documented MMR doses, and the remaining two patients had each received 1 dose. Of the 21 patients tested for mumps, 19 tested positive by reverse transcription–polymerase chain reaction assay or had a positive immunoglobulin M result. Nineteen of the 20 specimens tested with a mumps immunoglobulin G avidity assay had high-avidity antibodies; these cases were classified as secondary vaccine failures,* and one result was indeterminate.

During a widespread 2014 measles outbreak response in Kosrae, 4,360 MMR doses were administered (90% coverage of persons aged 6 months–57 years) (*3*). KDHS initially planned a similar mass MMR campaign for mumps outbreak control. However, review of vaccination records for the 21 mumps patients with ≥2 documented doses showed that 76.2% (95% confidence interval = 58%–94%; p<0.001) had received their last MMR dose before the 2014 campaign. Among these patients, the median interval since the last dose was 12 years. Investigations of recent mumps outbreaks suggest that waning of vaccine-induced immunity might contribute to transmission in populations with high MMR vaccination coverage (*4*). The current findings suggested that the 2014 MMR dose might have prevented additional mumps cases and that another mass vaccination activity was not warranted. Therefore, KDHS modified its initial response plan to a catch-up vaccination campaign for persons aged 1–24 years with <2 documented MMR doses.

KDHS declared the end of the outbreak on December 15, 2017. Unlike mumps in other Pacific Island communities, this outbreak remained small. The analysis suggests that the interval since last MMR dose contributed to mumps acquisition, and the 2014 campaign dose of MMR might have prevented further spread. Active case-finding and assessment of vaccination status enabled KDHS to save an estimated 1,000 MMR doses. This investigation underscored the importance of an accurate public health assessment of persons at risk for mumps to determine the most efficient and cost-effective outbreak response.

## Footnotes

* Secondary vaccine failure refers to waning of vaccine-induced immunity to nonprotective levels. Although distinguishing between primary and secondary vaccine failure is difficult, detection of mumps antibody with high avidity in a person with mumps suggests secondary failure.
